# Maize quality detection based on MConv-SwinT high-precision model

**DOI:** 10.1371/journal.pone.0312363

**Published:** 2025-01-24

**Authors:** Ning Zhang, Yuanqi Chen, Enxu Zhang, Ziyang Liu, Jie Yue

**Affiliations:** Engineering Research Center of Hydrogen Energy Equipment& Safety Detection, Universities of Shaanxi Province, Xijing University, Xi’an, China; Shijiazhuang Tiedao University, CHINA

## Abstract

The traditional method of corn quality detection relies heavily on the subjective judgment of inspectors and suffers from a high error rate. To address these issues, this study employs the Swin Transformer as an enhanced base model, integrating machine vision and deep learning techniques for corn quality assessment. Initially, images of high-quality, moldy, and broken corn were collected. After preprocessing, a total of 20,152 valid images were obtained for the experimental samples. The network then extracts both shallow and deep features from these maize images, which are subsequently fused. Concurrently, the extracted features undergo further processing through a specially designed convolutional block. The fused features, combined with those processed by the convolutional module, are fed into an attention layer. This attention layer assigns weights to the features, facilitating accurate final classification. Experimental results demonstrate that the MC-Swin Transformer model proposed in this paper significantly outperforms traditional convolutional neural network models in key metrics such as accuracy, precision, recall, and F1 score, achieving a recognition accuracy rate of 99.89%. Thus, the network effectively and efficiently classifies different corn qualities. This study not only offers a novel perspective and technical approach to corn quality detection but also holds significant implications for the advancement of smart agriculture.

## 1. Introduction

Wheat, maize, and rice are the world’s leading staple cereals, each cultivated on approximately 200 million hectares (rounded) [1]. Corn, a crucial staple in China, plays a vital role in ensuring national food security [2]. It serves as food for humans and animals and as a raw material in the industrial production of oil and anhydrous ethanol. During the harvest period, the moisture content of corn is usually around 25% [3]. This high grain moisture content can lead to a high rate of grain breakage during harvesting, which not only increases harvesting costs but also lowers grain quality, resulting in significant economic losses [4]. Furthermore, due to its inherent high moisture, large embryo, and strong hygroscopicity, corn kernels are prone to mildew during storage and transport, seriously affecting their quality and posing health threats to humans and animals [5]. Clearly, corn breakage and mold impact not only farmers’ incomes but also public health and industrial safety. Therefore, there is an urgent need for an accurate and efficient method for corn quality detection.

Traditionally, grain quality inspection involves manual sampling by workers and chemical analysis [6, 7]. However, this method is time-consuming, inefficient, and heavily influenced by the subjective judgment of inspectors. Moreover, chemical tests are costly, require dedicated facilities, and are destructive to the samples. Current quality detection methods for major food crops include near-infrared spectroscopy, hyperspectral and multispectral imaging, and deep learning-based approaches [8]. For instance, Yao et al. [9] employed a fluorescence hyperspectral imaging system using maximum likelihood and binary coding to detect aflatoxins in contaminated corn, achieving accuracies of 80% and 88% respectively, effectively identifying moldy corn. Rathna Priya et al. [10] utilized near-infrared hyperspectral imaging technology to detect corn grain quality, with detection accuracies for fungal infections ranging from 91% to 95%, achieving high precision. Han et al. [11] used two high-performance cameras with a dual violet external excitation source and new image processing software to capture fluorescence images of corn for aflatoxin detection. The normalized fluorescence index (NDFI) was used for automatic detection of contaminated seeds, achieving high sensitivity (0.987) and specificity (0.96) across a wide range. Despite these advancements, the high cost of equipment limits their practical application.

Recently, machine vision has rapidly advanced, with increasing research into its use for detecting and classifying corn quality based on the extraction of color, texture, and other features in corn grains. For example, Bi et al. [12] improved the Swin Transformer to classify corn seeds by collecting images of 19 different types and achieved high classification accuracy, providing valuable information for future seed classification development. Wang et al. [13] extracted geometric features of corn through machine vision, fine-tuned the model’s positive sample matching strategy using YOLO v7, and incorporated transformer encoder block modules and a coordinated attention mechanism, achieving a classification accuracy of 96.9%. Another study by Wang et al. [14] combined multispectral camera imaging with the watershed algorithm and a convolutional neural network model incorporating VGG16 and ResNet50 to predict the quality of corn seeds, including detection of insect pests, mold, and mechanical damage. The final accuracy was 95.63%, showing promise for classifying corn seed quality.

With its powerful learning ability and wide coverage, deep learning [15, 16] has made continuous breakthroughs in image fields such as object detection [17–19], image classification [20–22] and semantic segmentation [23–25]. Broken corn and intact corn have highly similar characteristics, while the spatial perception of CNN is localized, and the remote dependence relationship in the image cannot be modeled, and the ability to extract similar features is limited, and the accurate classification of corn seeds cannot be realized. Therefore, in order to identify different quality corn grains more accurately, a corn quality classification model based on Swin Transformer is proposed in this study. The model uses the self-attention mechanism to extract image information effectively, thus improving the overall extraction of maize seed features. By improving the features of corn seeds and images, the model can effectively analyze the subtle differences between different categories, and provide a new idea for the quality classification of corn grains.

## 2. Materials and methods

### 2.1. Image acquisition

**Fig 1:**
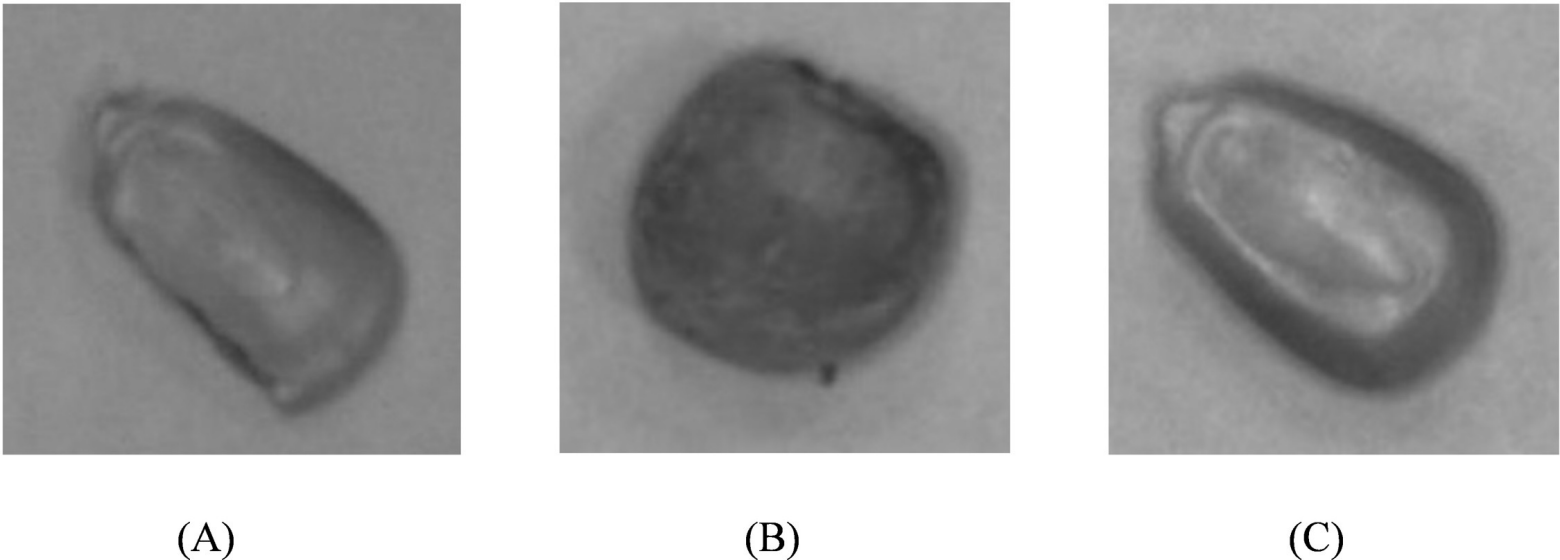
Gray-scale map of corn with different qualities. (A): good (B): mul (C): broken.

**Fig 2:**
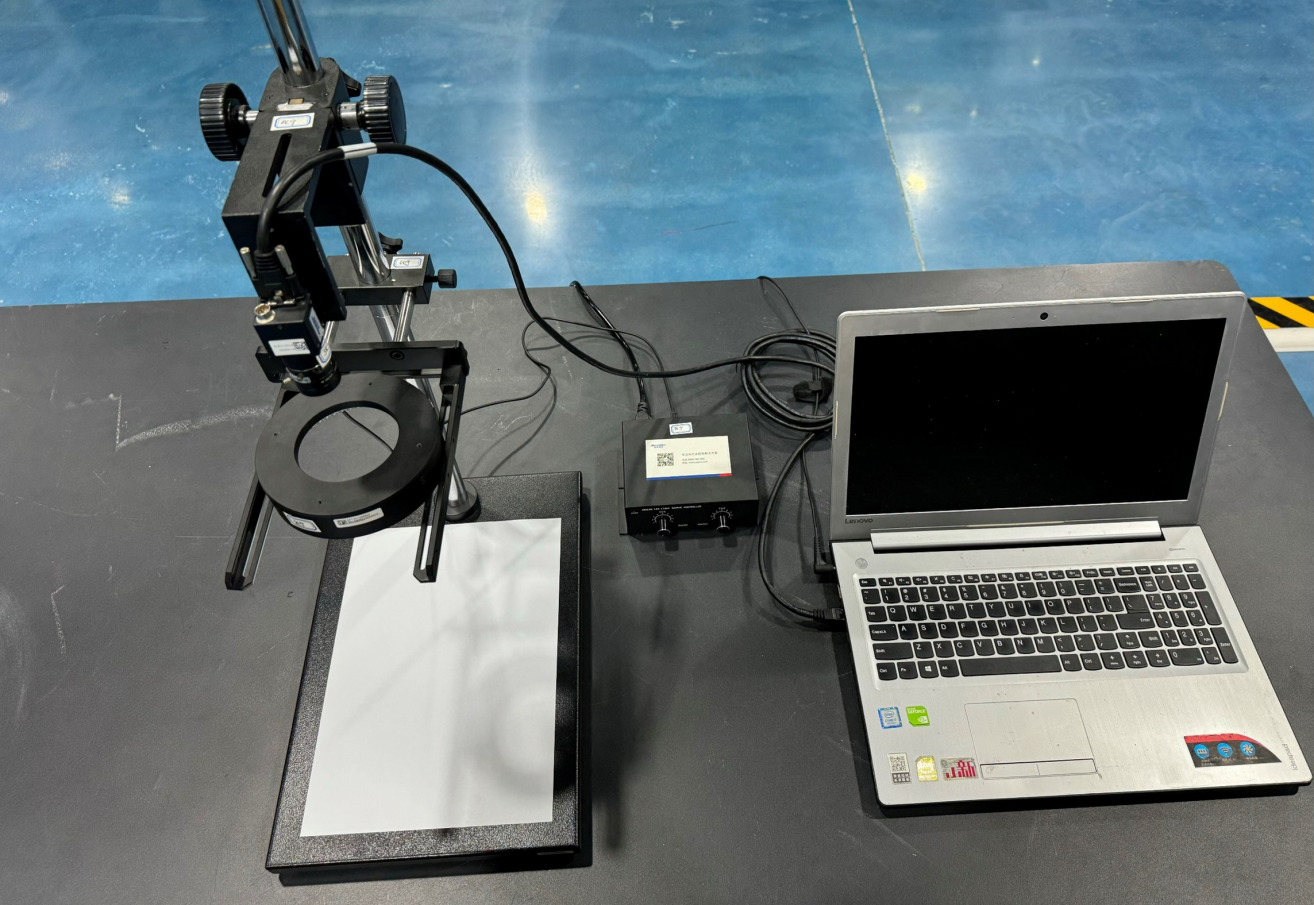
Maize image acquisition system.

Mouldy and broken kernels represent the primary defects in corn. To investigate these defects in greater detail, this study focused on a single variety of corn, capturing images of this variety in three distinct states of quality: good, mouldy, and broken. Fig 1 displays a grayscale image of the corn variety used in this study, highlighting the detailed features necessary for defect analysis. To mitigate any potential interference from variations in photographic equipment or lighting conditions, the same set of equipment and uniform lighting conditions were employed throughout the image capture process. This approach was critical in maintaining the integrity of the images and ensuring that any observed defects were attributable to the corn kernels themselves rather than inconsistencies in the imaging setup. Details of the photographic setup, including the camera specifications, lighting arrangements, and environmental controls, are illustrated in Fig 2.

### 2.2. Image preprocessing

**Fig 3:**
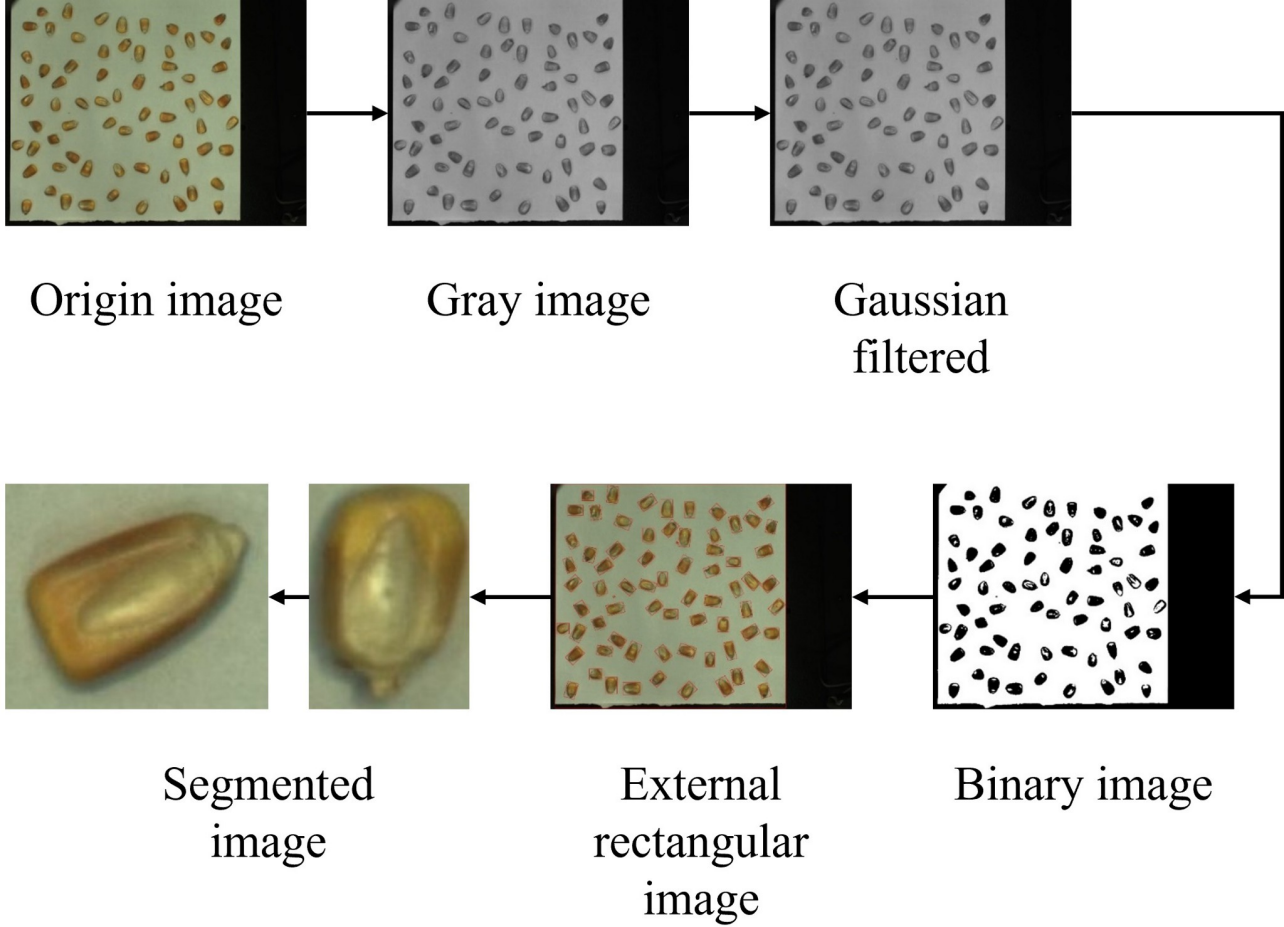
Corn image preprocessing process.

In the image preprocessing stage, to better identify the defects of individual corn kernels, we processed the original image containing a lot of corn, as shown in Fig 3. First, we performed granitization on the image and removed noise through Gaussian filtering [26] to enhance the clarity and quality of the image. Next, we applied thresholding [27] to binarize the image and extracted the contours to obtain the edge information of the corn kernels. By further processing, we obtained the coordinates of the center point for each kernel and cropped out the individual corn images based on the smallest external rectangle. Ultimately, we processed a total of 7,361 corn kernel images.

After image preprocessing, we successfully filtered various quality corn images, including 1,266 good quality corn images, 938 moldy corn images, and 1,157 broken corn images. However, statistical results indicate that the total number of samples is clearly insufficient, and the distribution of various corn images is uneven. In particular, the number of good corn images significantly exceeds that of defective corn, which may adversely affect the accuracy and stability of subsequent model classification [28, 29].

**Fig 4:**
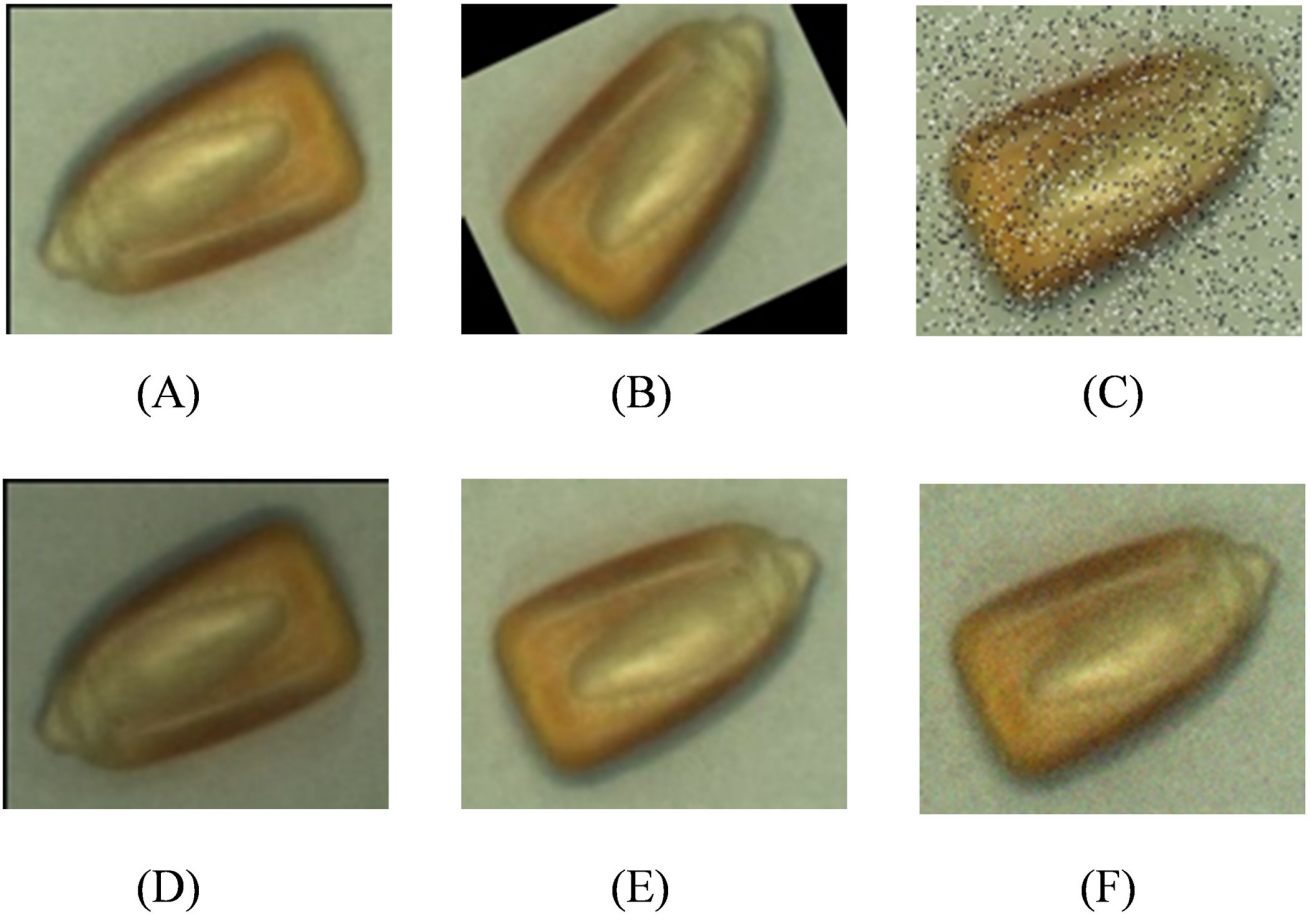
Image enhancement effect (A) Original image (B) random Angle inversion (C) salt and pepper noise (D) random brightness (E)180° rotation (F) Gaussian noise.

To address this shortcoming and improve the generalization ability and robustness of the model, we adopted a variety of data augmentation techniques. Specifically, we used random angle rotation to simulate corn images taken from different shooting angles, adjusted random brightness to simulate image performance under varying lighting conditions, and added salt-and-pepper noise and Gaussian blur processing methods to simulate possible image distortion and blurriness in real environments. These methods not only enriched the diversity of the dataset but also effectively increased the number of broken and moldy corn images, thus providing a more comprehensive and balanced training sample for the model, as shown in Fig 4.

By applying the described data augmentation techniques, we successfully expanded the original dataset from 3,361 images to a significantly larger set of 20,152 images. The expanded dataset is presented in Table 1. Notably, this augmented dataset is well-balanced across different categories of corn images. The dataset was divided into training and test sets following a 4:1 ratio, ensuring that the majority of the data is used for training purposes while a smaller portion is reserved for testing and evaluation. It is important to note that the data division was performed once and was not re-validated to avoid potential data leakage or overfitting issues.

**Table 1:** Sample size.

Maize category	Original quantity	Enhanced quantity
**Gc**	1266	6843
**Mc**	938	6566
**Bc**	1157	6743

Gc: Good corn; Mc: Moldy corn; Bc: Broken corn

### 2.3. MConv-SwinT model design concept

**Fig 5:**
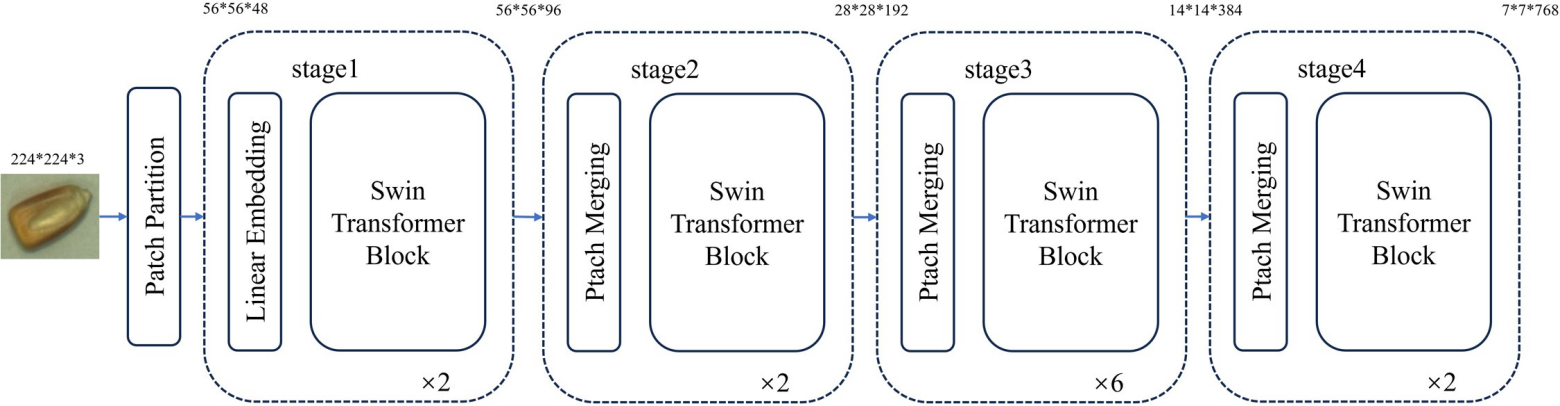
Main architecture of Swin Transformer.

With the development of research, many scholars have begun to apply Transformer technology in the field of computer vision. Unlike traditional convolutional neural networks (CNNS), Transformer’s self-attention mechanism can overcome the limitations of local interactions, which allows the model to be trained in parallel, and can effectively mine long-distance correlations in images to extract more powerful features. As a pioneer in introducing Transformer to replace conventional convolution operations in machine Vision, Vision Transformer (ViT) [30, 31] model shows certain advantages, but it still has shortcomings in processing spatial information modeling, large consumption of computing resources and low learning efficiency on small-scale data. Swin Transformer [32, 33] is an improved model proposed by Microsoft to address the limitations of Transformer model in the field of computer vision. It adopts a hierarchical construction method, which not only inherits the advantages of convolutional neural networks in processing large-size images, but also establishes long-distance dependence by moving Windows, effectively solving the problems of high computational complexity and insufficient information interaction between groups. Its structure is shown in Fig 5.

Low-order features refer to the fundamental characteristics of an object or scene that are directly observable and relatively easy to extract or perceive from an image. In contrast, higher-order features are more complex and arise from the detailed analysis and interpretation of lower-order features or their combinations. However, the Swin Transformer model has two key limitations. First, it heavily relies on the higher-order features from the final stage, often neglecting the changes in lower-order features present in earlier stages. Although these lower-order features are still in the preliminary processing phase, their ability to generalize across the entire corn image is limited due to the constraints of the current receptive field. Nonetheless, distinctions between high-quality corn and broken, moldy corn are not only based on high-order characteristics such as shape, texture, contour size, color, and light intensity, but also on low-order features. Second, a pure Transformer model requires a substantial number of training samples and lacks spatial inductive bias. To address these issues, we propose a new model, MConv-SwinT, which integrates shallow and deep features to enhance performance with a limited number of samples. This approach aims to more effectively capture and distinguish between different types of corn.

### 2.4. MConv-SwinT model

**Fig 6:**
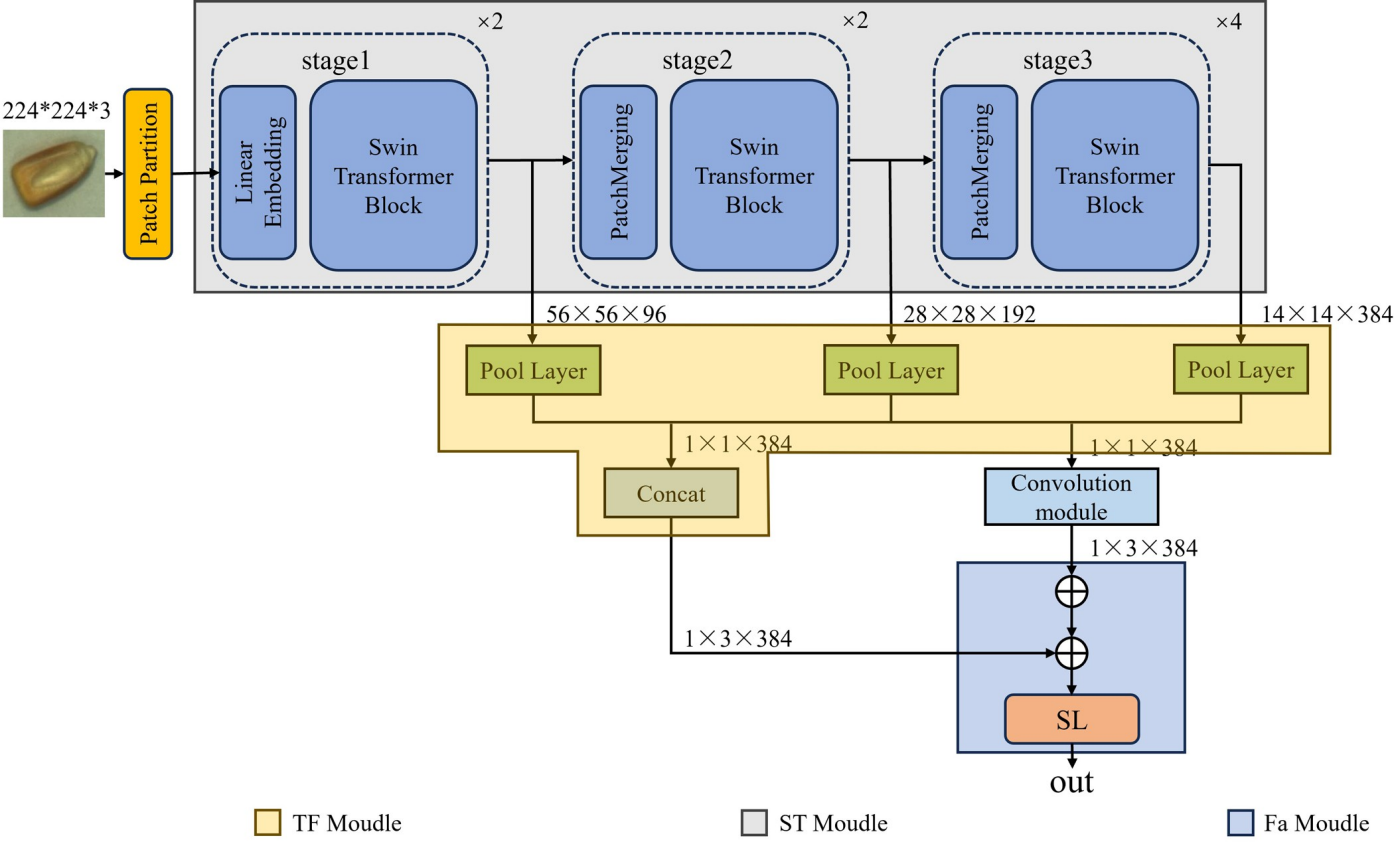
MConv-SwinT structure diagram.

The structure of the MConv-SwinT network designed in this paper is illustrated in Fig 6. The Swin Transformer initially comprises four stages. However, due to the increasing focus on multiple attention heads [34], the rise in channel numbers consumes substantial computational resources, making it challenging for machines with limited capabilities to meet the real-time corn detection requirements. To prevent overfitting resulting from excessive and complex parameter calculations, we removed the fourth stage of the Swin Transformer and reduced the number of blocks in the third stage to four. This adjustment significantly lowers the number of parameters and reduces detection time. We introduced the TF Module (Transformer Feature Fusion Module) to extract feature variables from each stage’s output and perform concatenation to obtain features enriched with information across all dimensions of the image. Concurrently, these feature vectors are passed to the Convolution Module to capture spatial dimension information. The processed information is then combined through an addition operation and forwarded to the Fa Module (Feature Attention Module) to produce the final output.

#### 2.4.1. TF module

The image I ∈ RC×H×W is inputted into the network, and the feature vector Fx ∈ RCx×Hx×Wx passed through each stage is obtained through calculation, where: C, H, and W respectively represent the input height, width, and number of channels of the image; Cx, Hx, and Wx respectively represent the height, width, and number of channels of each stage feature. Specifically, if the input image I ∈ R224×224×3 is sent to the network, the output size of the first stage is F1 ∈ R56×56×96, the output size of the second stage is F2 ∈ R28×28×192, and the output size of the third stage is F3 ∈ R14×14×384. Subsequently, the feature vector output of each stage is passed as its input to each subsequent stage, where the feature vector output of stage e1 is passed to stage e2 and stage e3. The feature vector from the stage 2 output is passed to stage 3. Finally, a Pool layer consisting of Linear, GELU, LayerNorm, and Pool is added at each stage. An adaptive pooling layer is employed for downsampling, and the dimensionality of feature vectors is unified, ensuring that the dimensionality of feature vectors output in the three stages is consistent. In other words, based on F3 ∈ R14×14×384, the dimensions are unified into F1, 2, and 3 ∈ R1×1×384. Finally, on the one hand, we utilize the Concat method to concatenate the feature vectors of different stages. The calculation formula of feature fusion vector is shown in formula 1.


F4=Concat(F1,F2,F3) (1)

Where F4∈R1×3×384 represents the dimension obtained by concatenating the feature vectors of the three stages.

#### 2.4.2. Convolution module

**Fig 7:**
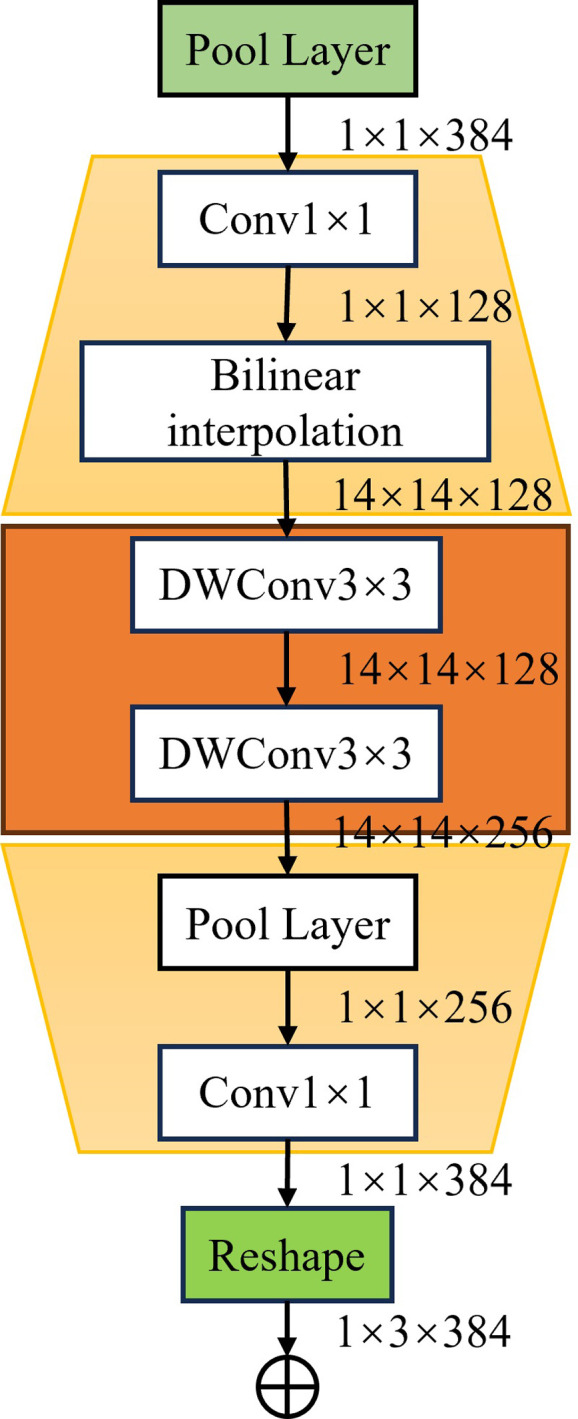
Feature attention module.

We designed a convolutional module that takes as input the feature vectors obtained after average pooling. Depth-separable convolution primarily reduces the number of parameters required for convolution by decoupling the spatial and channel dimensions, thereby improving the efficiency of convolutional kernels. Given that the input feature vector has dimensions of 1×1×384 and cannot utilize convolutional kernels larger than 1×1, we first apply a 1×1 convolutional kernel to reduce the channel dimensions from 1×1×384 to 1×1×128. Next, we extend the feature map by spatially upsampling to a size of 14×14, which is large enough to accommodate 3×3 convolution. We then use two consecutive 3×3 depth-separable convolutions to capture more complex spatial features. Following this, we apply global average pooling to reduce the spatial dimensions from 14×14 to 1×1, resulting in a feature vector of dimensions 1×1×256. We then use a 1×1 convolution to increase the dimensions from 1×1×256 to 1×1×1152, and finally reshape the feature vector to 1×3×384. The feature vector F4, obtained from concatenation, is then added to the feature vector F5 from the convolutional module to produce the final vector F6. The integration of convolutional neural networks significantly enhances the Transformer model’s ability to handle small amounts of data, while the inclusion of depth-separable convolutions adds only a minimal number of parameters. The resulting feature vector F6, with its multi-scale representation, contains richer spatial and channel information, thereby enhancing feature expression. Additionally, this residual-like structure helps mitigate the vanishing gradient problem, facilitating the training of deeper networks. The convolutional module structure is shown in Fig 7.

#### 2.4.3. Fa module

**Fig 8:**
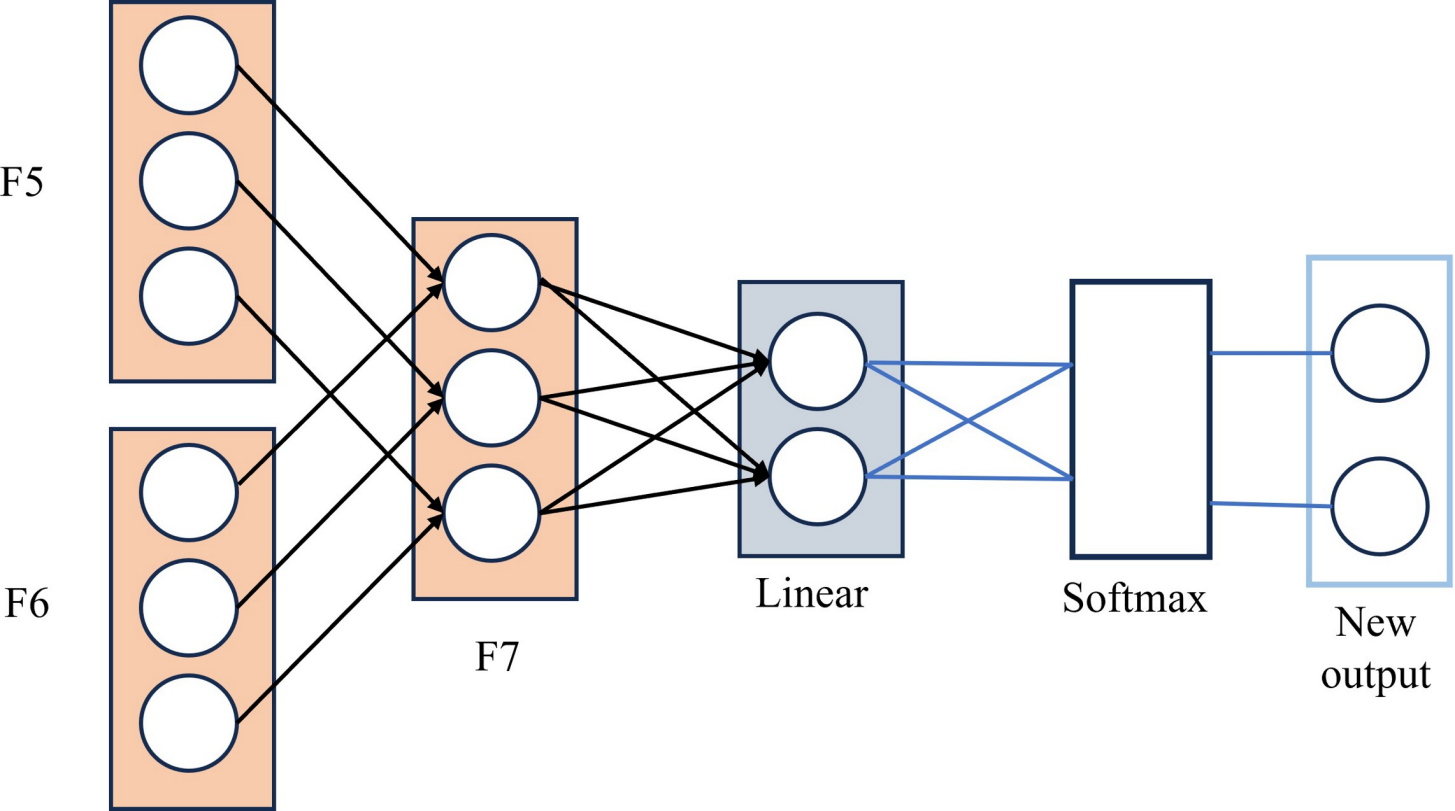
Feature attention module.

Subsequently, this paper designs a feature attention layer SL, which is composed of Linear layer and Softmax in Fig 8.

Softmax calculation formula is as follows:

$$Soft\;\max(z_{i})=\frac{\exp(z_{i})}{\sum_{j}\exp(z_{j})}$$ (2)

Input the spliced feature vectors into the feature attention layer, assign different weights to the feature vectors at different stages, and get w1, w2 and w3, so that important features are given greater weights to achieve enhancement, while other features are given smaller weights to achieve autonomous suppression, to learn the key features of different corn seed varieties. Multiply the weights and vectors to get the final output.

Finally, F7 was input into SoftMax classifier to classify and identify corn of different quality. The specific calculation is as follows:

Out=Softmax(Linear(F7)) (3)

## 3. Results

### 3.1. Experimental parameter

The classification model and comparison experiment proposed in this paper were carried out under Windows operating environment, and the experimental model was implemented by Pytorch deep learning framework. The specific experimental environment parameters are shown in Table 2.

**Table 2:** Operating environment parameters.

A	System	Df	Language	CUDA	GPU	Vm(G)
Parameter	Windows	Pytorch1. 13. 1	Python3. 9. 12	11. 6	3060	12

As: Accessories; Df: Development framework; Vm: Video memory

Swin Transformer model is used to run several times under different parameters, and a better model is obtained. First, we set the size of the input corn seed image to 224×224. Learning rate is an important parameter. If the learning rate is too large, the model will not converge, while if the learning rate is too small, the training speed will be greatly slowed down. Extend your training time. Therefore, we set the learning rate of three groups to 0.001,0.0001 and 0.00001 for testing during training, and we set the batch size to 8,16 and 32 respectively. In the training process, we found that the model could converge when the epoch was lower than 60, so we set the epoch to 60 and used the AutoAugment [35] method to enhance the data set to improve the generalization of the model and avoid overfitting. Finally, the parameters we selected were tested, as shown in Table 3.

**Table 3:** Model parameters.

Parameter	Value
**Epoch**	60
**Learning rate**	0. 0001
**Batch size**	32

### 3.2. Evaluation index

In the field of machine learning, the confusion matrix is an essential tool for evaluating model classification performance in supervised learning. This matrix is structured simply: each column represents a category predicted by the model, while each row represents the actual category. Considering the common binary classification problem, we can clearly define four types of classification outcomes: when both the actual and predicted results are Positive, it is termed True Positive (TP); when the actual result is Negative but the predicted result is Positive, it is False Positive (FP). Conversely, when the actual result is Positive but the predicted result is Negative, it is False Negative (FN); and when both the actual and predicted results are Negative, it is called True Negative (TN). The specific structure of the confusion matrix is depicted in Table 4, which clearly illustrates the distribution of these four outcomes, thereby aiding in the comprehensive evaluation of the model’s classification performance.

**Table 4:** Confusion matrix for binary classification problem.

CM		Pr	Nr
**Forecast**	**Positive**	TP	FP
**Results**	**Negative**	FN	TN

CM: Confusion Matrix; Pr: Positive result; Nr: Negative result.

For the corn dataset constructed in this study, we employed a confusion matrix to evaluate the performance of each network model in recognizing different qualities of corn. We calculated accuracy, precision, recall, and F1 scores, treating each category as “positive” and all others as “negative” to precisely gauge the model’s ability to recognize each corn type. To comprehensively assess network performance, we computed the accuracy and recall rates across the three corn types. Accuracy and recall rates often present a trade-off. To address this, we introduced the F1 score as a comprehensive metric, calculating the weighted accuracy and recall rates, thus providing a balanced and thorough evaluation of model performance. A higher F1 score indicates better model balance between accuracy and recall, leading to superior performance. The detailed processes for calculating these evaluation metrics are depicted in Table 5.

**Table 5:** Parameter calculation formulas.

Index	Formula	Significance
**Accuracy**	$Accuracy=\frac{TP+TN}{TP+TN+FP+FN}$	Correctly estimate the number as a percentage of the total
**Precision**	$\mathrm{Pr}ecision=\frac{TP_{i}}{TP_{i}+FP_{i}}$	The probability that all samples predicted by the model to be positive examples are actually positive examples
**Recall**	Recall=TPiTPi+FNi	The probability that the model predicts correctly in all positive cases
**F1-Score**	$F1=2\times \frac{\text{Re}call\times \text{Pr}ecision}{\text{Re}call+\text{Pr}ecision}$	F1 score takes into account both recall and accuracy

### 3.3. Formatting of mathematical components

#### 3.3.1. Feature extraction experiment

Swin transformer model is used in this experiment. First, maize seed images were fed into the network to obtain 7×7 depth features of 768 channels. Then, the probability of the three categories is predicted through the classification layer. Feature extraction experiment is the basic experiment of this paper. Finally, as shown in Table 6, the accuracy on the maize image dataset was 98.50%.

**Table 6:** Confusion matrix for binary classification problem.

EI	Baseline	TF	TF + FA
**Accuracy (%)**	98. 50	99. 49	99. 89
**Precision (%)**	98. 50	99. 48	99. 83
**Recall (%)**	98. 46	99. 49	99. 87
**F1-Score (%)**	98. 48	99. 48	99. 84

EI: Evaluation Indicators.

#### 3.3.2 Ablation experiment

Firstly, feature fusion module and neural network architecture are introduced to extract key information from features of three different stages effectively through automatic average pooling. Then, these extracted features are fused and spliced, and then the features are input into the bottleneck block, and the output features and fusion features are added to generate new output features. The experimental results are shown in Table 6. On the corn data set, the accuracy of the model reaches 99.49%. Compared with the original model, the accuracy of the model is improved by 0.99%, which highlights the effectiveness of the feature fusion module in improving the accuracy of the model.

#### 3.3.3 Multi-scale feature fusion experiment with feature attention layer

Subsequently, we introduced new attention mechanisms and processed the fused features in detail. The effect of this newly added attention mechanism is that it can more accurately screen out features that are critical to model decisions. Through this process, we successfully obtain the final multi-scale fusion feature. The results of the experiment are shown in Table 6. The data show that compared with the experiment of 3. 3. 2, the accuracy of our model in the task of corn classification has reached 99.89%, an increase of 0.4%. This remarkable progress fully demonstrates the effectiveness of the newly added attention mechanism in extracting key features and improving the classification ability of the model.

#### 3.3.4 Contrast test

To verify the effectiveness and superiority of the improved model proposed in this paper, MConv-SwinT model is compared with some representative convolutional neural networks under the same data set, experimental environment, and network parameter configuration. Including VGG16, Resnet50, Densnet121, Vision Transformer, Swin Transformer models, MobileNet V2, MobileViT V2, Deit3 and EffcientNet V2.

**Fig 9:**
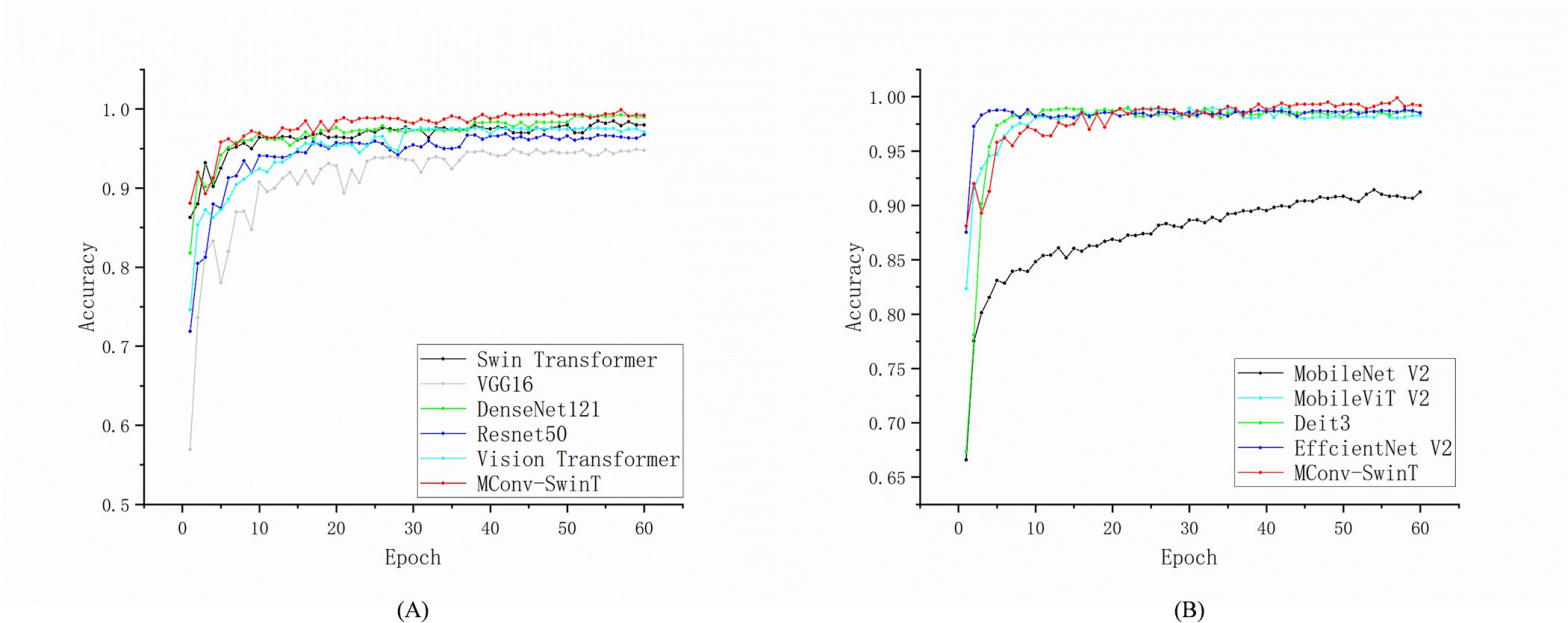
Accuracy curves of all models in corn dataset.

Fig 9 shows the relationship between the accuracy curve and the number of training iterations. The validation accuracy curve provides an indicator to evaluate the performance of the model. By observing the change of the curve, we can know whether the classification accuracy of the model on the verification set increases, remains stable or decreases with the progress of training. And by observing the verification accuracy curve, we can identify whether the model has overfitting or underfitting problems. It can be clearly found in the figure that the MConv-SwinT model curve keeps rising with the increase of iterations and becomes stable at 20 epochs, indicating that the model has no overfitting problem. The maximum accuracy of the model is 99.89%, which is obviously higher than other models, indicating that the model has good recognition accuracy and generalization ability.

#### 3.3.5 Significance test

To evaluate the performance advantages of our proposed MConv-SwinT model compared to other advanced models, we conducted a series of statistical significance tests. We employed the paired t-test method, which is particularly suitable for comparing the performance of different models on the same dataset. The paired t-test considers the differences between each pair of observations, thus providing a more precise comparison. We compared the MConv-SwinT model with nine mainstream deep learning models, including MobileNet V2, MobileViT V2, Deit3, EfficientNet V2, Swin Transformer, VGG16, DenseNet121, Resnet50, and Vision Transformer. For each model, we collected accuracy data from 60 rounds of training, ensuring a sufficiently large sample size to obtain reliable statistical results.

In our significance tests, we used three key indicators: p-value, t-statistic, and confidence interval. The p-value represents the probability of observing the current or more extreme results, assuming the null hypothesis is true. In our study, a p-value less than 0.05 was considered statistically significant, indicating that the performance difference between the MConv-SwinT model and the comparison model is unlikely to be caused by random factors. The t-statistic measures the size of the observed difference relative to its standard error. A larger absolute t-value indicates a more significant difference. A positive t-value indicates that the MConv-SwinT model performs better than the comparison model, while a negative value indicates the opposite. The 95% confidence interval provides an estimated range of the average performance difference between the MConv-SwinT model and the comparison model. If the confidence interval does not include 0, it further supports the conclusion that there is a significant difference. The specific results are shown in Table 7.

**Table 7:** Significance test results.

Master model	Contrast model	t-value	p-value	Confidence interval
**MConv-SwinT**	MobileNet V2	10.4886	4.17689E-15	(0.011018, 0.016212)
	MobileViT V2	11.7973	3.678E-17	(0.055039, 0.077523)
	Deit3	7.8571	9.54802E-11	(0.007073, 0.011907)
	EffcientNet V2	12.0651	1.433E-17	(0.029806, 0.041658)
	Swin Transformer	10.4886	4.17689E-15	(0.011018, 0.016212)
	VGG16	11.7973	3.678E-17	(0.055039, 0.077523)
	DenseNet121	7.8571	9.54802E-11	(0.007073, 0.011907)
	Resnet50	12.0651	1.433E-17	(0.029806, 0.041658)
	Vision Transformer	10.1644	1.39113E-14	(0.022872, 0.034085)

Based on our significance test results, we found that the MConv-SwinT model is statistically significantly superior to all compared models. All p-values for comparisons are far below 0.05 (with the largest being 9.54802e-11), strongly indicating that the observed performance differences are not coincidental. Compared to MobileViT V2 and VGG16, the MConv-SwinT model demonstrates the most substantial performance improvement. With a t-statistic of 11.7973 and a 95% confidence interval of (0.055039, 0.077523), this indicates that the model’s accuracy is on average 5.5% to 7.8% higher. Even for the closest performing models (Deit3 and DenseNet121), the MConv-SwinT model still maintains a significant advantage. The t-statistic of 7.8571 and a 95% confidence interval of (0.007073, 0.011907) show an average accuracy increase of 0.7% to 1.2%. For other models such as EfficientNet V2 and Resnet50, the model also demonstrates clear advantages, with an average accuracy improvement of 3% to 4.2% (t = 12.0651, CI: 0.029806 to 0.041658). These statistical results strongly support the model’s superiority across various comparison scenarios, not only surpassing all compared models in average accuracy but also showing that this advantage is statistically significant.

#### 3.3.6 Feature fusion experiment with feature attention layer

In this experiment, multi-scale features of maize seed images were extracted. In order to prevent overfitting due to too many parameters and improve model applicability, we have removed stage 4 from the Swin Transformer model. First, maize images are input into the network, and adaptive average pooling method is used to sample the features of the three stages, and the features are uniformly mapped to channel dimension 384. Then the features of the above three stages are spliced and average fused to obtain the multi-scale fusion features. The fusion vector is output to the feature attention layer to enhance the required features. Finally, the classifier is used for classification. The experimental accuracy is shown in Table 8, which lists the evaluation indicators of MConv-SwinT, Swin Transformer, VGG16, Resnet50 and DenseNet121. The Accuracy, Precision, Recall and F1-Score of MConv-SwinT are 99.89%, 99.83%, 99.87% and 99.84%, respectively. Better performance than other models.

**Table 8:** All model evaluation indicators.

Model	Accuracy(%)	Precision(%)	Recall(%)	F1-Score(%)
**VGG16**	94. 86	94. 86	94. 86	94. 86
**DenseNet121**	99.27	99.32	99.30	99.31
**Resnet50**	96.77	96.78	96.77	96.77
**Vision Transformer**	97.61	97.66	97.63	97.64
**MobileNet V2**	91.43	88.14	87.88	88.01
**MobileViT V2**	98.98	99.15	98.93	99.04
**Deit3**	98.81	98.85	98.93	99.89
**EffcientNet V2**	98.79	98.82	98.80	98.81
**Swin Transformer**	98. 50	98. 50	98. 46	98. 48
**MConv-SwinT**	99.89	99.83	99.87	99.85

#### 3.3.7 Confusion matrix

**Fig 10:**
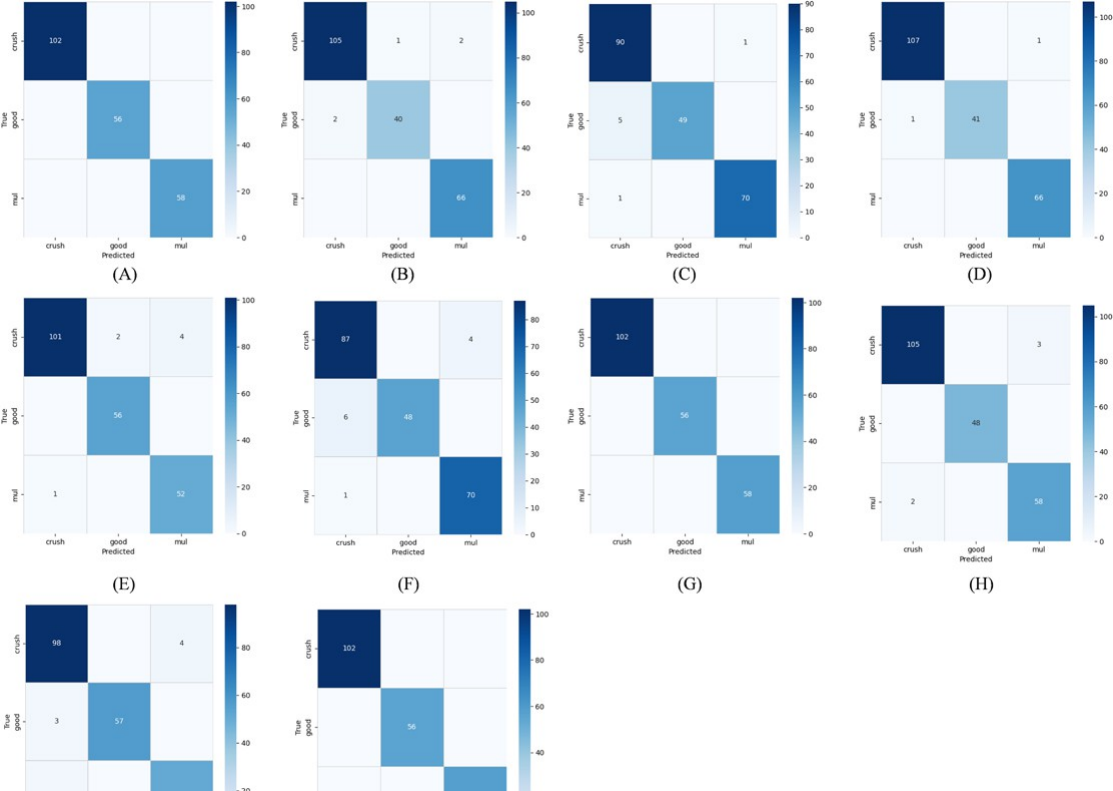
Confusion matrix graph (A) MobileViT V2; (B) Vision Transformer; (C) Deit3; (D) Swin Transformer; (E) Resnet50; (F) VGG16; (G) DenseNet121; () EffcientNet V2; (I) MobileNet V2;(J) MConv-SwinT. H.

To evaluate the performance of the network more comprehensively and deeply, the confusion matrix between MConv-SwinT network and comparison network is specially drawn in this paper, and the comparison analysis diagram is made. These charts not only visually show the actual recognition of different quality corn by MConv-SwinT network, but also further highlight its superiority in recognition ability by comparing with the comparison network. As shown in Fig 10, the MConv-SwinT network has shown higher accuracy in identifying corn quality, demonstrating its significant advantages in handling such tasks. Compared with traditional manual recognition methods, MConv-SwinT network not only has higher accuracy, but also shows more advantages in recognition speed, stability and the ability to handle large-scale data. These advantages make the application prospect of the network in the agricultural field very broad, is expected to provide more reliable and efficient technical support for corn quality identification.

### 3.4 Comparison experiment with different data sets

**Fig 11:**
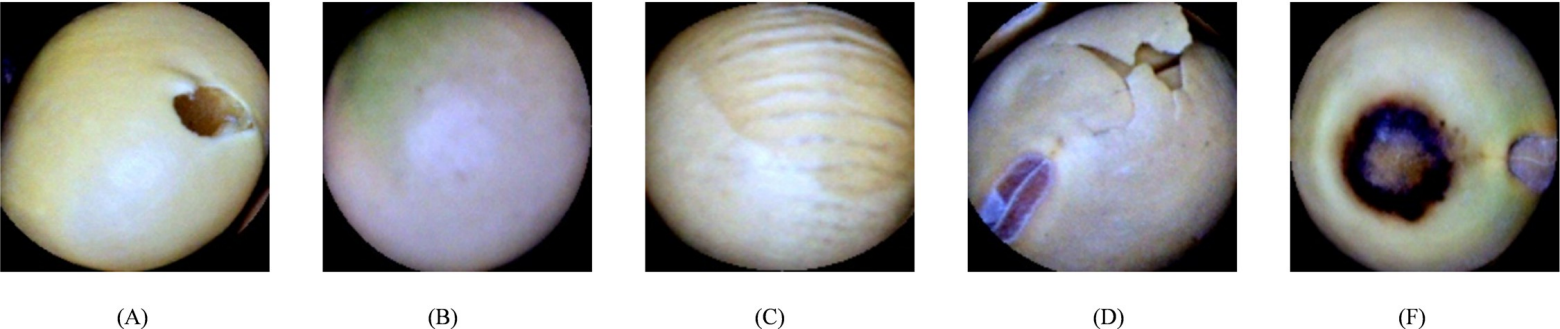
Soybean image (A): Broken; (B): Immature; (C): Intact; (D): Skin-damaged; (E): Spotted.

In order to prove the superiority of the model in quality detection, we selected the public soybean quality data set to test all models, and the soybean quality in the data set was divided into five categories, as shown in Fig 11.

At the same time, in order to test the performance of the model when the amount of data is small, we no longer carry out data balance on the data set, and the quantity of each type of soybean is shown in Table 9.

**Table 9:** Soybean sample size.

Type	Number
**Broken**	1002
**Immature**	1125
**Intact**	1201
**Skin-damaged**	1127
**Spotted**	1058

**Fig 12:**
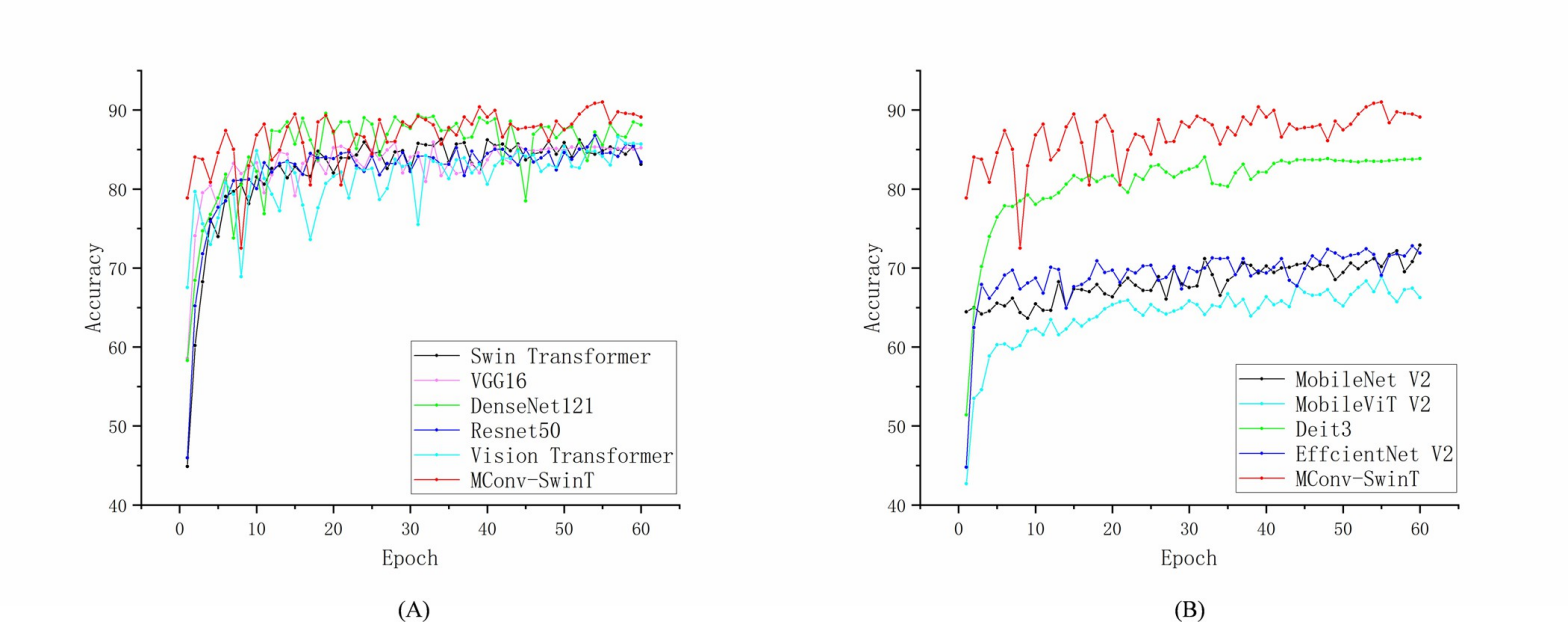
Accuracy curves of all models in soybean dataset.

The detection parameters are the same as in the original experiment. Fig 12 shows the change of the training accuracy curve and the comparison with other models. The results are shown in Table 9.

As shown in Table 10, this model achieves an accuracy rate of 91.02%, which is significantly higher than that of other leading models currently available. These results demonstrate the model’s strong generalization ability and outstanding performance in quality detection, with the fusion layer proving effective at capturing multi-scale spatial features. Additionally, the model excels in detecting small datasets, highlighting the effectiveness of our designed convolutional module with a bottleneck block structure in information extraction.

**Table 10:** Evaluation index comparison.

Model	Accuracy(%)	Precision(%)	Recall(%)	F1-Score(%)
**VGG16**	85.95	85.60	85.49	85.48
**DenseNet121**	89.57	89.73	89.23	89.40
**Resnet50**	86.76	86.27	86.27	86.25
**Vision Transformer**	86.58	86.38	86.58	86.23
**MobileNet V2**	72.89	72.79	72.27	72.36
**MobileViT V2**	68.90	69.34	68.38	68.63
**Deit3**	84.04	83.58	83.62	83.53
**EffcientNet V2**	78.80	72.73	72.31	72.42
**Swin Transformer**	86.22	86.43	85.84	85.91
**MConv-SwinT**	91.02	91.13	90.79	90.89

## 4. Discussion

This paper introduces an efficient and accurate model for recognizing corn quality. We began by constructing a dataset that includes various quality types of corn seeds, such as whole, moldy, and broken corn. By employing preprocessing and contour extraction techniques, we transformed complex corn images into clear single-core representations, laying a foundation for subsequent recognition tasks. To enhance the model’s stability and generalization, we implemented a data augmentation strategy. Given the subtle appearance differences among corn qualities and the importance of shallow features, we specifically designed a maize quality recognition model, MConv-SwinT, which leverages feature attention and multi-scale feature fusion. This model not only achieves fast processing speeds and minimal hardware requirements but also delivers high recognition accuracy, offering a powerful tool for corn quality detection in intelligent agriculture.

Although deep networks can capture the contours and shape features of corn, they often miss critical details and require extensive data for training. To address this issue, we integrated a feature attention mechanism and a multi-scale feature fusion strategy into the model. This approach enables the model to effectively capture multi-scale feature information and extract more comprehensive and detailed corn quality data. Furthermore, to prevent overfitting due to an excessive number of parameters, we truncated stage 4 in the backbone network and reduced stage 3 Block to 4. This adjustment not only simplifies the model but also enhances its practical performance. Notably, the implementation cost of this model is low, requiring only a standard digital camera for image capture, making it highly suitable for widespread adoption in smart agriculture. Compared to other classical network models, the proposed MConv-SwinT model demonstrates superior recognition capability and performance. The integration of deep learning technology enhances automatic feature extraction, avoiding the cumbersome manual feature extraction required by traditional methods.

However, this research is currently limited to evaluating different quality types within a single maize variety. Future work will focus on expanding the scope to include quality identification across various maize varieties. Additionally, to address potential issues such as pest infestations, we plan to enlarge our dataset and refine the model to meet a broader range of identification needs. Through continuous optimization and enhancement, we are committed to making further contributions to the advancement of smart agriculture.

## 5. Conclusions

This paper introduces an innovative method for corn quality recognition, which can efficiently and accurately classify images of corn seeds based on quality, addressing the high error rates and inefficiencies of traditional methods. The key findings of this study are detailed below:

Firstly, we collected image data of several corn varieties and employed grayscale processing, Gaussian filters, and minimum boundary matrices to reduce noise and segment the images, ensuring each image contained only one corn seed. Based on this, we constructed a maize dataset comprising three distinct quality categories with a total of 20,152 images, providing a substantial data resource for further research.Secondly, recognizing the importance of both shallow and deep features in corn quality assessment, we developed a neural network model, the MConv-SwinT, which utilizes feature attention and multi-scale feature fusion. This model effectively integrates shallow and deep features to enhance recognition capabilities. This innovative approach offers a novel strategy and technical solution for corn quality inspection.

In terms of experimental validation, we employed various evaluation metrics to comprehensively assess the model’s performance. The results indicate that the MConv-SwinT model significantly outperforms the baseline Swin Transformer model in terms of accuracy, precision, recall, and F1 score, achieving a recognition accuracy of 98.89%. Further, the model’s superiority was confirmed through ablation studies and confusion matrix analysis. Future research will focus on optimizing the model’s structure, enhancing its operational speed, and broadening its applicability to enable real-time, online identification of multiple corn qualities, thereby providing more effective and precise technical support for agricultural production.
